# (2-Acetyl­phenolato)(2,2′-bipyridine)nitratocopper(II)

**DOI:** 10.1107/S1600536809042718

**Published:** 2009-10-23

**Authors:** Li-Hua Chen, Gan-Qing Zhao, Xiang Wang, Qin-Long Peng, Zi-Hong Pan

**Affiliations:** a School of Chemistry and Chemical Engineering, Pingdingshan University, Pingdingshan 467000, People’s Republic of China

## Abstract

In the title compound, [Cu(C_8_H_7_O_2_)(NO_3_)(C_10_H_8_N_2_)], the Cu^II^ ion is five-coordinate in a distorted square-pyramidal geometry. The basal positions are occupied by two N atoms from a 2,2′-bipyridine ligand and two O atoms from the 2-acetyl­phenolate anion. The axial position is occupied by one O atom of a nitrate anion. In the bipyridine ligand, the two pyridine rings are slightly twisted by an angle of 3.5 (1)°. The crystal structure is stabilized by C—H⋯O hydrogen bonds

## Related literature

For related structures, see: Bevan *et al.* (1963 ▶); Falguni *et al.* (1998 ▶); Garland *et al.* (1986 ▶); Gasque *et al.* (1999 ▶); Ming *et al.* (1995 ▶); Reki *et al.* (1998 ▶); Solans *et al.* (1987 ▶). For the synthesis, see: Plesch *et al.* (1997 ▶).
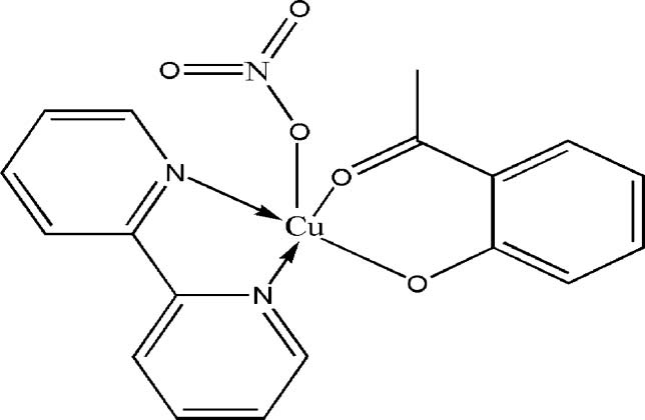


## Experimental

### 

#### Crystal data

[Cu(C_8_H_7_O_2_)(NO_3_)(C_10_H_8_N_2_)]
*M* *_r_* = 416.87
Monoclinic, 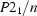

*a* = 13.4683 (12) Å
*b* = 8.3101 (8) Å
*c* = 15.5924 (15) Å
β = 108.583 (1)°
*V* = 1654.2 (3) Å^3^
*Z* = 4
Mo *K*α radiation
μ = 1.36 mm^−1^
*T* = 296 K
0.30 × 0.30 × 0.20 mm

#### Data collection

Bruker APEXII CCD area-detector diffractometer
Absorption correction: multi-scan (*SADABS*; Sheldrick, 1996 ▶) *T*_min_ = 0.686, *T*_max_ = 0.773
8261 measured reflections
2917 independent reflections
2370 reflections with *I* > 2σ(*I*)
*R*_int_ = 0.025

#### Refinement

*R*[*F* ^2^ > 2σ(*F* ^2^)] = 0.029
*wR*(*F* ^2^) = 0.074
*S* = 1.03
2917 reflections
245 parameters
H-atom parameters constrained
Δρ_max_ = 0.22 e Å^−3^
Δρ_min_ = −0.27 e Å^−3^

### 

Data collection: *APEX2* (Bruker, 2008 ▶); cell refinement: *SAINT* (Bruker, 2008 ▶); data reduction: *SAINT*; program(s) used to solve structure: *SHELXS97* (Sheldrick, 2008 ▶); program(s) used to refine structure: *SHELXL97* (Sheldrick, 2008 ▶); molecular graphics: *SHELXTL* (Sheldrick, 2008 ▶); software used to prepare material for publication: *SHELXTL*.

## Supplementary Material

Crystal structure: contains datablocks global, I. DOI: 10.1107/S1600536809042718/ci2918sup1.cif

Structure factors: contains datablocks I. DOI: 10.1107/S1600536809042718/ci2918Isup2.hkl

Additional supplementary materials:  crystallographic information; 3D view; checkCIF report

## Figures and Tables

**Table 1:** Selected bond lengths (Å)

Cu1—O1	1.8832 (17)
Cu1—O2	1.9307 (17)
Cu1—N2	1.9941 (19)
Cu1—N1	1.998 (2)
Cu1—O3	2.434 (2)

**Table 2:** Hydrogen-bond geometry (Å, °)

*D*—H⋯*A*	*D*—H	H⋯*A*	*D*⋯*A*	*D*—H⋯*A*
C2—H2⋯O3^i^	0.93	2.59	3.401 (4)	146
C7—H7⋯O3^ii^	0.93	2.51	3.343 (3)	150

Symmetry codes: (i) 
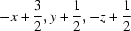
; (ii) 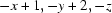.

**Fig. 1.**
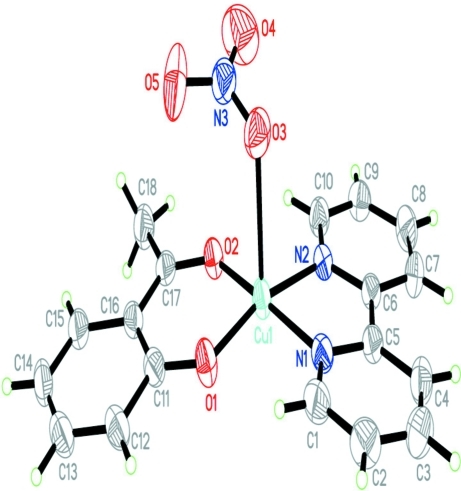
The molecular structure of the title compound, showing 50% probability displacement ellipsoids and the atom-numbering scheme.

## supplementary crystallographic information

### Comment

Crystal structures of bis(salicylaldehydato)copper(II) (Bevan *et al.*, 1963), (1,10-phenanthroline)(salicylaldehydato)copper(II) (Solans *et al.*, 1987), Aqua(4,7-diphenyl-1,10-phenanthroline)(salicylaldehydato)copper(II) nitrate monohydrate (Gasque *et al.*, 1999), (2,2^'^-bipyridine)(salicylaldehydato)copper(II) (Garland *et al.*, 1986), [Cu(5-carborxysalicylaldehyde)(2,2^'^-bipyridine)(ClO_4_)] and [Cu(5-carborxysalicylaldehyde)(2,2^'^-bipyridine)(2H_2_O)] (Reki *et al.*, 1998), [Cu(salicylaldehyde)(1,10-phenanthroline)(ClO_4_)]_2_ (Ming *et al.*, 1995). We report here the crystal structure of the title Cu^II^ complex.

In the title compound, the Cu^II^ ion is in a distorted square-pyramidal geometry (Fig. 1 and Table 1). The four basal positions are occupied by two N donor atoms from a 2,2^'^-bipyridine ligand and two O atoms from the 2-acetylphenolate anion. The axial position is occupied by one O atom of a nitrate anion. The Cu1 atom is displaced from the O1/O2/N1/N2 basal plane toward the O3 atom by 0.1472 (3) Å. In the pipyridine ligand, the two pyridine rings are twisted slightly by an angle of 3.5 (1)°. The N1- and N2-pyridine rings form dihedral angles of 16.2 (1) and 15.6 (1)°, respectively, with the benzene ring.

The crystal structure is stabilized by C—H···O hydrogen bonds (Table 2).

### Experimental

The crystal used in this structure determination was obtained adventitiously from an attempted preparation of a copper(II)-Schiff base complex. It was synthesized as described in the literature (Plesch *et al.*, 1997). 2-Hydroxyacetophenone (1.00 mmol) in methanol (10 ml) was added dropwise to a soltion of beta-alaine (1.00 mmol) and potassium hydroxide (1.00 mmol) in methanol (10 ml). The yellow solution was stirred for 2 h at 333 K. The resultant mixture was added dropwise to copper(II) nitrate trihydrate (1.00 mmol) and 2,2^'^-bipyridine (1.00 mmol) in an aqueous methanolic solution (20 ml, 1:1 *v*/*v*), and heated with stirring for 2 h at 333 K. The dark blue solution obtained was filtered and left for several days; dark blue crystals were formed which were filtered off, washed with water, and dried under vacuum.

### Refinement

H atoms were positioned geometrically and refined as riding, with C-H = 0.93 (CH) or 0.96 Å (CH_3_) and *U*_iso_(H) = x*U*_eq_(C,N), where x = 1.5 for methyl H atoms and 1.2 for other H atoms.

### Crystal data

**Table:**

[Cu(C_8_H_7_O_2_)(NO_3_)(C_10_H_8_N_2_)]	*F*(000) = 852
*M**_r_* = 416.87	*D*_x_ = 1.674 Mg m^−^^3^
Monoclinic, *P*2_1_/*n*	Mo *K*α radiation, λ = 0.71073 Å
Hall symbol: -P 2yn	Cell parameters from 3127 reflections
*a* = 13.4683 (12) Å	θ = 2.4–27.0°
*b* = 8.3101 (8) Å	µ = 1.36 mm^−^^1^
*c* = 15.5924 (15) Å	*T* = 296 K
β = 108.583 (1)°	Block, dark green
*V* = 1654.2 (3) Å^3^	0.30 × 0.30 × 0.20 mm
*Z* = 4	

### Data collection

**Table:**

Bruker APEXII CCD area-detector diffractometer	2917 independent reflections
Radiation source: fine-focus sealed tube	2370 reflections with *I* > 2σ(*I*)
graphite	*R*_int_ = 0.025
φ and ω scans	θ_max_ = 25.1°, θ_min_ = 2.4°
Absorption correction: multi-scan (*SADABS*; Sheldrick, 1996)	*h* = −15→15
*T*_min_ = 0.686, *T*_max_ = 0.773	*k* = −9→7
8261 measured reflections	*l* = −18→17

### Refinement

**Table:**

Refinement on *F*^2^	Primary atom site location: structure-invariant direct methods
Least-squares matrix: full	Secondary atom site location: difference Fourier map
*R*[*F*^2^ > 2σ(*F*^2^)] = 0.029	Hydrogen site location: inferred from neighbouring sites
*wR*(*F*^2^) = 0.074	H-atom parameters constrained
*S* = 1.03	*w* = 1/[σ^2^(*F*_o_^2^) + (0.0308*P*)^2^ + 0.9795*P*] where *P* = (*F*_o_^2^ + 2*F*_c_^2^)/3
2917 reflections	(Δ/σ)_max_ = 0.001
245 parameters	Δρ_max_ = 0.22 e Å^−^^3^
0 restraints	Δρ_min_ = −0.27 e Å^−^^3^

### Special details

**Table:**

Geometry. All esds (except the esd in the dihedral angle between two l.s. planes) are estimated using the full covariance matrix. The cell esds are taken into account individually in the estimation of esds in distances, angles and torsion angles; correlations between esds in cell parameters are only used when they are defined by crystal symmetry. An approximate (isotropic) treatment of cell esds is used for estimating esds involving l.s. planes.
Refinement. Refinement of *F*^2^ against ALL reflections. The weighted *R*-factor *wR* and goodness of fit *S* are based on *F*^2^, conventional *R*-factors *R* are based on *F*, with *F* set to zero for negative *F*^2^. The threshold expression of *F*^2^ > σ(*F*^2^) is used only for calculating *R*-factors(gt) *etc*. and is not relevant to the choice of reflections for refinement. *R*-factors based on *F*^2^ are statistically about twice as large as those based on *F*, and *R*- factors based on ALL data will be even larger.

### Fractional atomic coordinates and isotropic or equivalent isotropic displacement parameters (Å^2^)

**Table:**

	*x*	*y*	*z*	*U*_iso_*/*U*_eq_	
Cu1	0.77943 (2)	0.96237 (4)	0.03090 (2)	0.03038 (11)	
C1	0.7404 (2)	1.1660 (3)	0.17445 (18)	0.0395 (7)	
H1	0.8040	1.1268	0.2122	0.047*	
C2	0.6852 (2)	1.2727 (4)	0.20915 (19)	0.0453 (7)	
H2	0.7101	1.3038	0.2696	0.054*	
C3	0.5919 (2)	1.3328 (4)	0.1521 (2)	0.0473 (7)	
H3	0.5538	1.4070	0.1735	0.057*	
C4	0.5554 (2)	1.2825 (3)	0.06330 (19)	0.0400 (7)	
H4	0.4924	1.3216	0.0245	0.048*	
C5	0.61386 (18)	1.1732 (3)	0.03286 (16)	0.0300 (6)	
C6	0.58213 (18)	1.1050 (3)	−0.05922 (16)	0.0292 (6)	
C7	0.4907 (2)	1.1450 (3)	−0.12742 (17)	0.0377 (6)	
H7	0.4449	1.2210	−0.1174	0.045*	
C8	0.4688 (2)	1.0699 (3)	−0.21052 (18)	0.0418 (7)	
H8	0.4079	1.0951	−0.2572	0.050*	
C9	0.5378 (2)	0.9575 (3)	−0.22366 (17)	0.0376 (6)	
H9	0.5242	0.9058	−0.2791	0.045*	
C10	0.6270 (2)	0.9231 (3)	−0.15362 (17)	0.0352 (6)	
H10	0.6733	0.8467	−0.1623	0.042*	
C11	0.99646 (19)	0.9179 (3)	0.13299 (17)	0.0324 (6)	
C12	1.08113 (19)	0.9544 (3)	0.21194 (18)	0.0378 (6)	
H12	1.0700	1.0212	0.2559	0.045*	
C13	1.1790 (2)	0.8929 (4)	0.2243 (2)	0.0441 (7)	
H13	1.2334	0.9192	0.2764	0.053*	
C14	1.1984 (2)	0.7920 (3)	0.1607 (2)	0.0443 (7)	
H14	1.2647	0.7482	0.1711	0.053*	
C15	1.11933 (19)	0.7571 (3)	0.08252 (19)	0.0384 (6)	
H15	1.1331	0.6919	0.0392	0.046*	
C16	1.01667 (18)	0.8190 (3)	0.06647 (17)	0.0306 (6)	
C17	0.93694 (19)	0.7830 (3)	−0.01883 (17)	0.0309 (6)	
C18	0.9599 (2)	0.6816 (3)	−0.08916 (19)	0.0445 (7)	
H18A	0.8996	0.6788	−0.1425	0.067*	
H18B	0.9769	0.5743	−0.0664	0.067*	
H18C	1.0182	0.7265	−0.1039	0.067*	
N1	0.70583 (15)	1.1164 (2)	0.08838 (13)	0.0309 (5)	
N2	0.64940 (15)	0.9956 (2)	−0.07351 (13)	0.0285 (5)	
N3	0.73727 (19)	0.5847 (3)	0.03372 (17)	0.0450 (6)	
O1	0.90495 (13)	0.9788 (2)	0.12828 (12)	0.0381 (4)	
O2	0.84484 (12)	0.8358 (2)	−0.04004 (11)	0.0332 (4)	
O3	0.70722 (15)	0.7105 (2)	0.06448 (14)	0.0491 (5)	
O4	0.6824 (2)	0.5290 (3)	−0.03905 (18)	0.0818 (8)	
O5	0.8215 (2)	0.5239 (3)	0.07571 (19)	0.0848 (9)	

### Atomic displacement parameters (Å^2^)

**Table:**

	*U*^11^	*U*^22^	*U*^33^	*U*^12^	*U*^13^	*U*^23^
Cu1	0.02264 (17)	0.0360 (2)	0.03004 (18)	0.00320 (14)	0.00488 (12)	−0.00188 (14)
C1	0.0357 (15)	0.0422 (16)	0.0360 (15)	−0.0034 (12)	0.0049 (12)	−0.0056 (12)
C2	0.0476 (18)	0.0519 (18)	0.0375 (16)	−0.0052 (15)	0.0152 (14)	−0.0125 (14)
C3	0.0493 (18)	0.0474 (18)	0.0527 (19)	0.0033 (14)	0.0266 (15)	−0.0135 (14)
C4	0.0335 (15)	0.0429 (16)	0.0458 (17)	0.0060 (13)	0.0156 (13)	0.0001 (13)
C5	0.0266 (13)	0.0314 (14)	0.0328 (14)	0.0008 (11)	0.0106 (11)	0.0004 (11)
C6	0.0250 (13)	0.0294 (13)	0.0342 (14)	0.0003 (11)	0.0107 (11)	0.0023 (11)
C7	0.0310 (14)	0.0416 (16)	0.0384 (15)	0.0110 (12)	0.0078 (12)	0.0040 (12)
C8	0.0319 (15)	0.0528 (19)	0.0341 (15)	0.0050 (13)	0.0010 (12)	0.0070 (13)
C9	0.0374 (15)	0.0428 (16)	0.0290 (14)	0.0006 (13)	0.0056 (11)	−0.0032 (12)
C10	0.0315 (14)	0.0380 (15)	0.0350 (15)	0.0028 (12)	0.0093 (11)	−0.0061 (11)
C11	0.0268 (14)	0.0333 (14)	0.0343 (14)	−0.0015 (11)	0.0058 (11)	0.0095 (11)
C12	0.0303 (14)	0.0448 (17)	0.0347 (15)	−0.0050 (13)	0.0052 (11)	0.0065 (12)
C13	0.0304 (15)	0.0487 (17)	0.0450 (17)	−0.0069 (13)	0.0006 (12)	0.0133 (14)
C14	0.0233 (14)	0.0453 (17)	0.0602 (19)	0.0046 (12)	0.0076 (13)	0.0179 (15)
C15	0.0283 (14)	0.0352 (15)	0.0513 (17)	0.0021 (12)	0.0122 (13)	0.0079 (13)
C16	0.0254 (13)	0.0304 (14)	0.0345 (14)	0.0004 (11)	0.0073 (11)	0.0066 (11)
C17	0.0285 (14)	0.0269 (13)	0.0382 (14)	0.0010 (11)	0.0120 (11)	0.0064 (11)
C18	0.0360 (15)	0.0458 (17)	0.0486 (17)	0.0083 (13)	0.0094 (13)	−0.0072 (14)
N1	0.0260 (11)	0.0327 (12)	0.0322 (12)	−0.0015 (9)	0.0068 (9)	−0.0027 (9)
N2	0.0228 (11)	0.0312 (12)	0.0303 (12)	−0.0001 (9)	0.0066 (9)	−0.0001 (9)
N3	0.0406 (14)	0.0425 (15)	0.0541 (16)	0.0045 (12)	0.0183 (12)	0.0111 (12)
O1	0.0268 (10)	0.0503 (12)	0.0340 (10)	0.0036 (8)	0.0052 (8)	−0.0058 (8)
O2	0.0252 (9)	0.0395 (10)	0.0334 (10)	0.0030 (8)	0.0070 (7)	−0.0010 (8)
O3	0.0493 (12)	0.0463 (12)	0.0590 (13)	0.0069 (10)	0.0275 (10)	0.0012 (10)
O4	0.0774 (18)	0.088 (2)	0.0722 (18)	−0.0149 (15)	0.0133 (15)	−0.0282 (15)
O5	0.0627 (16)	0.094 (2)	0.091 (2)	0.0436 (15)	0.0165 (15)	0.0261 (16)

### Geometric parameters (Å, °)

**Table:**

Cu1—O1	1.8832 (17)	C9—H9	0.93
Cu1—O2	1.9307 (17)	C10—N2	1.332 (3)
Cu1—N2	1.9941 (19)	C10—H10	0.93
Cu1—N1	1.998 (2)	C11—O1	1.313 (3)
Cu1—O3	2.434 (2)	C11—C16	1.416 (4)
C1—N1	1.338 (3)	C11—C12	1.419 (3)
C1—C2	1.374 (4)	C12—C13	1.369 (4)
C1—H1	0.93	C12—H12	0.93
C2—C3	1.381 (4)	C13—C14	1.386 (4)
C2—H2	0.93	C13—H13	0.93
C3—C4	1.378 (4)	C14—C15	1.370 (4)
C3—H3	0.93	C14—H14	0.93
C4—C5	1.381 (4)	C15—C16	1.421 (3)
C4—H4	0.93	C15—H15	0.93
C5—N1	1.350 (3)	C16—C17	1.450 (3)
C5—C6	1.474 (3)	C17—O2	1.256 (3)
C6—N2	1.351 (3)	C17—C18	1.492 (4)
C6—C7	1.387 (3)	C18—H18A	0.96
C7—C8	1.383 (4)	C18—H18B	0.96
C7—H7	0.93	C18—H18C	0.96
C8—C9	1.379 (4)	N3—O5	1.224 (3)
C8—H8	0.93	N3—O4	1.230 (3)
C9—C10	1.371 (3)	N3—O3	1.269 (3)
			
O1—Cu1—O2	92.60 (7)	O1—C11—C16	125.4 (2)
O1—Cu1—N2	167.80 (8)	O1—C11—C12	116.5 (2)
O2—Cu1—N2	92.87 (8)	C16—C11—C12	118.1 (2)
O1—Cu1—N1	92.18 (8)	C13—C12—C11	120.9 (3)
O2—Cu1—N1	171.30 (8)	C13—C12—H12	119.5
N2—Cu1—N1	81.10 (8)	C11—C12—H12	119.5
O1—Cu1—O3	101.92 (8)	C12—C13—C14	121.2 (3)
O2—Cu1—O3	86.63 (7)	C12—C13—H13	119.4
N2—Cu1—O3	89.28 (7)	C14—C13—H13	119.4
N1—Cu1—O3	99.49 (7)	C15—C14—C13	119.6 (3)
N1—C1—C2	122.4 (3)	C15—C14—H14	120.2
N1—C1—H1	118.8	C13—C14—H14	120.2
C2—C1—H1	118.8	C14—C15—C16	121.1 (3)
C1—C2—C3	118.3 (3)	C14—C15—H15	119.4
C1—C2—H2	120.8	C16—C15—H15	119.4
C3—C2—H2	120.8	C11—C16—C15	119.0 (2)
C4—C3—C2	119.8 (3)	C11—C16—C17	122.2 (2)
C4—C3—H3	120.1	C15—C16—C17	118.8 (2)
C2—C3—H3	120.1	O2—C17—C16	123.4 (2)
C3—C4—C5	119.0 (3)	O2—C17—C18	115.0 (2)
C3—C4—H4	120.5	C16—C17—C18	121.6 (2)
C5—C4—H4	120.5	C17—C18—H18A	109.5
N1—C5—C4	121.1 (2)	C17—C18—H18B	109.5
N1—C5—C6	114.3 (2)	H18A—C18—H18B	109.5
C4—C5—C6	124.6 (2)	C17—C18—H18C	109.5
N2—C6—C7	120.9 (2)	H18A—C18—H18C	109.5
N2—C6—C5	114.7 (2)	H18B—C18—H18C	109.5
C7—C6—C5	124.4 (2)	C1—N1—C5	119.3 (2)
C8—C7—C6	118.8 (2)	C1—N1—Cu1	125.73 (18)
C8—C7—H7	120.6	C5—N1—Cu1	114.92 (16)
C6—C7—H7	120.6	C10—N2—C6	119.7 (2)
C9—C8—C7	119.5 (2)	C10—N2—Cu1	125.48 (17)
C9—C8—H8	120.2	C6—N2—Cu1	114.78 (16)
C7—C8—H8	120.2	O5—N3—O4	121.3 (3)
C10—C9—C8	118.9 (3)	O5—N3—O3	119.4 (3)
C10—C9—H9	120.5	O4—N3—O3	119.2 (3)
C8—C9—H9	120.5	C11—O1—Cu1	127.05 (16)
N2—C10—C9	122.1 (2)	C17—O2—Cu1	129.18 (16)
N2—C10—H10	118.9	N3—O3—Cu1	115.43 (16)
C9—C10—H10	118.9		

### Hydrogen-bond geometry (Å, °)

**Table:**

*D*—H···*A*	*D*—H	H···*A*	*D*···*A*	*D*—H···*A*
C2—H2···O3^i^	0.93	2.59	3.401 (4)	146
C7—H7···O3^ii^	0.93	2.51	3.343 (3)	150

Symmetry codes: (i) −*x*+3/2, *y*+1/2, −*z*+1/2; (ii) −*x*+1, −*y*+2, −*z*.
